# Treatment of Cystitis

**Published:** 1881-06

**Authors:** 


### TREATMENT OF CYSTITIS.

Much difficulty is sometimes experienced in the treatment of this affection. Dr. A. J. C. Skene, of Brooklyn, gives the following which he regards as almost specific in its influence, especially in the earlier stages, affording rapid and lasting relief:

℞ Acidi Benzoici.
Sodii Biboratis *aa* grs. x.
Inf. Buchu ℥ij —M.

Sig.—This quantity to be taken three or four times a day. The diet should also be carefully regulated and the skin and bowels kept in an active condition.—*Canada Lancet.*

